# Australian research priorities for inherited retinal diseases: a James Lind Alliance priority setting partnership

**DOI:** 10.1136/bmjopen-2025-100301

**Published:** 2025-09-30

**Authors:** Eden G Robertson, Kate Hetherington, Meredith Prain, Alan Ma, Lauren N Ayton, Robyn V Jamieson, Emily Shepard, Leighton Boyd, Julia Hall, Rosemary Boyd, Sally Karandrews, Hollie Feller, Matthew P Simunovic, John R Grigg, Kanae Yamamoto, Claire E Wakefield, Anai Gonzalez-Cordero

**Affiliations:** 1 School of Clinical Medicine, UNSW Medicine and Health, University of New South Wales, Sydney, New South Wales, Australia; 2 Behavioural Sciences Unit, Kids Cancer Centre, Sydney Children’s Hospitals Network Randwick, Randwick, New South Wales, Australia; 3 Stem Cell Medicine Group, Children’s Medical Research Institute, Westmead, New South Wales, Australia; 4 Able Australia Services, Melbourne, Victoria, Australia; 5 University of School of Health Sciences, Audiology and Speech Pathology, The University of Melbourne, Parkville Campus, Melbourne, Victoria, Australia; 6 Eye Genetics Research Unit, Children’s Medical Research Institute, The University of Sydney, Sydney, New South Wales, Australia; 7 The Sydney Children’s Hospitals Network, Westmead, New South Wales, Australia; 8 Specialty of Genomic Medicine, Faculty of Medicine and Health, The University of Sydney, Sydney, New South Wales, Australia; 9 Departments of Optometry and Vision Sciences, and Surgery (Ophthalmology), The University of Melbourne, Parkville Campus, Melbourne, Victoria, Australia; 10 Centre for Eye Research Australia, Royal Victorian Eye and Ear Hospital, East Melbourne, Victoria, Australia; 11 Department of Clinical Genetics, The Sydney Children’s Hospitals Network, Westmead, New South Wales, Australia; 12 Eye Genetics Research Unit, Children’s Medical Research Institute, Westmead, New South Wales, Australia; 13 UsherKids Australia, Melbourne, Victoria, Australia; 14 Murdoch Children’s Research Institute, Parkville, Victoria, Australia; 15 Retina Australia, Melbourne, Victoria, Australia; 16 Blind Citizens Australia, Sydney, New South Wales, Australia; 17 Save Sight Institute, Faculty of Medicine and Health, The University of Sydney, Sydney, New South Wales, Australia; 18 Sydney Hospital and Sydney Eye Hospital, Sydney, New South Wales, Australia; 19 N/A, N/A, N/A, Australia; 20 School of Medical Sciences, Faculty of Medicine and Health, The University of Sydney, Sydney, New South Wales, Australia

**Keywords:** Community Participation, OPHTHALMOLOGY, Community-Based Participatory Research, Person-Centered Care, Rare Diseases, Research Design

## Abstract

**Abstract:**

**Objectives:**

Inherited retinal diseases (IRDs) are a broad range of diseases associated with abnormalities/degeneration of retinal cells. We aimed to identify the top 10 Australian research priorities for IRDs to ultimately facilitate more meaningful and potentially cost-effective research.

**Design:**

We conducted a James Lind Alliance priority setting partnership that involved two Australian-wide surveys and online workshops.

**Setting:**

Australia-wide.

**Participants:**

Individuals aged 16 years or older were eligible to participate if they had an IRD, were caregivers of an individual with an IRD or were health professionals providing care to this community.

**Outcome measure:**

In Survey 1, we gathered participants’ unanswered questions about IRDs. We grouped these into summary questions and undertook a literature review to verify if they were truly unanswered (ie, evidence uncertainties). In Survey 2, participants voted for the uncertainties that they considered a priority. Top-ranked uncertainties progressed for discussion and final prioritisation in two workshops.

**Results:**

In Survey 1, we collected 223 questions from 69 participants. We grouped these into 42 summary questions and confirmed 41 as evidence uncertainties. In Survey 2, 151 participants voted, with the 16 uncertainties progressing to final prioritisation. The top 10 priorities, set by the 24 workshop participants, represented (1) treatment/cure; (2) symptoms and disease progression; (3) psychosocial well-being and (4) health service delivery. The #1 priority was for treatment to prevent, slow down or stop vision loss, followed by the #2 priority to address the psychological impact of having an IRD.

**Conclusion:**

The top 10 research priorities highlight the need for IRD research that takes a whole-person, systems approach. Collaborations to progress priorities will accelerate the translation of research into real-world benefits.

**STRENGTHS AND LIMITATIONS OF THIS STUDY**

- This was the first James Lind Alliance priority setting partnership conducted in Australia or worldwide for inherited retinal diseases (IRDs).
- We embedded accessible research practices to ensure that individuals who have a vision impairment were able to participate.
- Our study included a broad representation of IRDs; however, it may have had an over-representation of individuals with milder levels of vision impairment.
- Our study eligibility limited the inclusion of individuals who may be linguistically diverse and those children/young people under 16 years of age.
- We did not measure the intersectionality of participants (eg, individuals who are deafblind), which may have influenced priorities.

## Introduction

 Inherited retinal diseases (IRDs) represent a broad range of genetic eye diseases that are associated with abnormalities or degeneration of retinal cells. They are genetically and phenotypically heterogeneous, with more than 320 causative genes identified to date.^1^ In Australia, IRDs are the leading cause of blindness in adults of working age and the second most common cause of blindness in children.^2^ While there are limited data on the prevalence of IRDs in Australia, current estimates sit between 1 in 1000 and 1 in 3000.^3 4^ With the overall lifetime cost per person with an IRD calculated to be $A5.2 million, the combined cost of IRDs to the Australian community and health system is immense.^5^

The heterogeneity of IRDs and the ongoing nature of progression mean that disease management is extremely complex. The Royal Australian and New Zealand College of Ophthalmologists (RANZCO) highlights the multidisciplinary approach required to manage the complex medical, psychosocial and practical challenges of living with an IRD.^6^ In 2020, a gene therapy for biallelic *RPE65*-associated retinal dystrophy (‘Luxturna’) was approved for therapeutic use in Australia.^7^ Aside from Luxturna, there are no other treatments clinically available for individuals with an IRD. With limited treatment options, most individuals with an IRD will progressively lose their vision. Over time, declining vision may reduce quality of life, with challenges including managing education and career paths, living independently, navigating daily life and relationships.^8^,^12^

Across most health research in Australia and internationally, the research agenda is typically set by funding bodies and researchers.^13 14^ This can result in a disconnect between the research agendas set forth and what is most important to those who will be most impacted by this research—including patients, caregivers and health professionals.^15^ Disconnects in research agendas can be costly, especially with much research lost to the translational pipeline gap.^16^ Identifying consumers’ research priorities in a structured, unbiased manner ensures the opportunity to align research investment with consumer needs, enhancing the likelihood of translation and thus research impact.

The James Lind Alliance is a non-profit initiative that brings patients, carers and clinicians together to undertake priority setting partnerships (PSPs).^17^ Priority setting partnerships facilitate the identification and prioritisation of evidence uncertainties, or ‘unanswered research questions’, that stakeholders agree are the most important for research to address. The purpose is to inform future research agendas so that research is as meaningful and impactful as possible. In 2012, a James Lind Alliance PSP was undertaken in the UK to preventiondiagnose and treat sight loss and eye conditions.^18^ Their initial survey gathered uncertainties from any individual who had been or may be affected by sight loss and relevant health professionals, with the interim prioritisation and final workshops conducted separately for the IRD community. No PSP has yet been undertaken that focuses solely on IRDs, that also includes psychosocial-related uncertainties or that is within the Australian context. Since 2012, there have been significant advancements in gene identification, diagnostic yield for individuals with a suspected IRD, cell biology techniques and emerging therapies for IRDs. Building on the 2012 PSP, we aimed to identify the top 10 research priorities for IRDs in Australia, from the perspectives of those with lived experience, and providing healthcare for this community was considered most important.

## Methods and materials

We undertook a James Lind Alliance PSP to achieve our aim. Our protocol can be found online.^19^ This PSP is reported according to the reporting guideline for priority setting of health research guidelines.^20^

**Figure 1:**
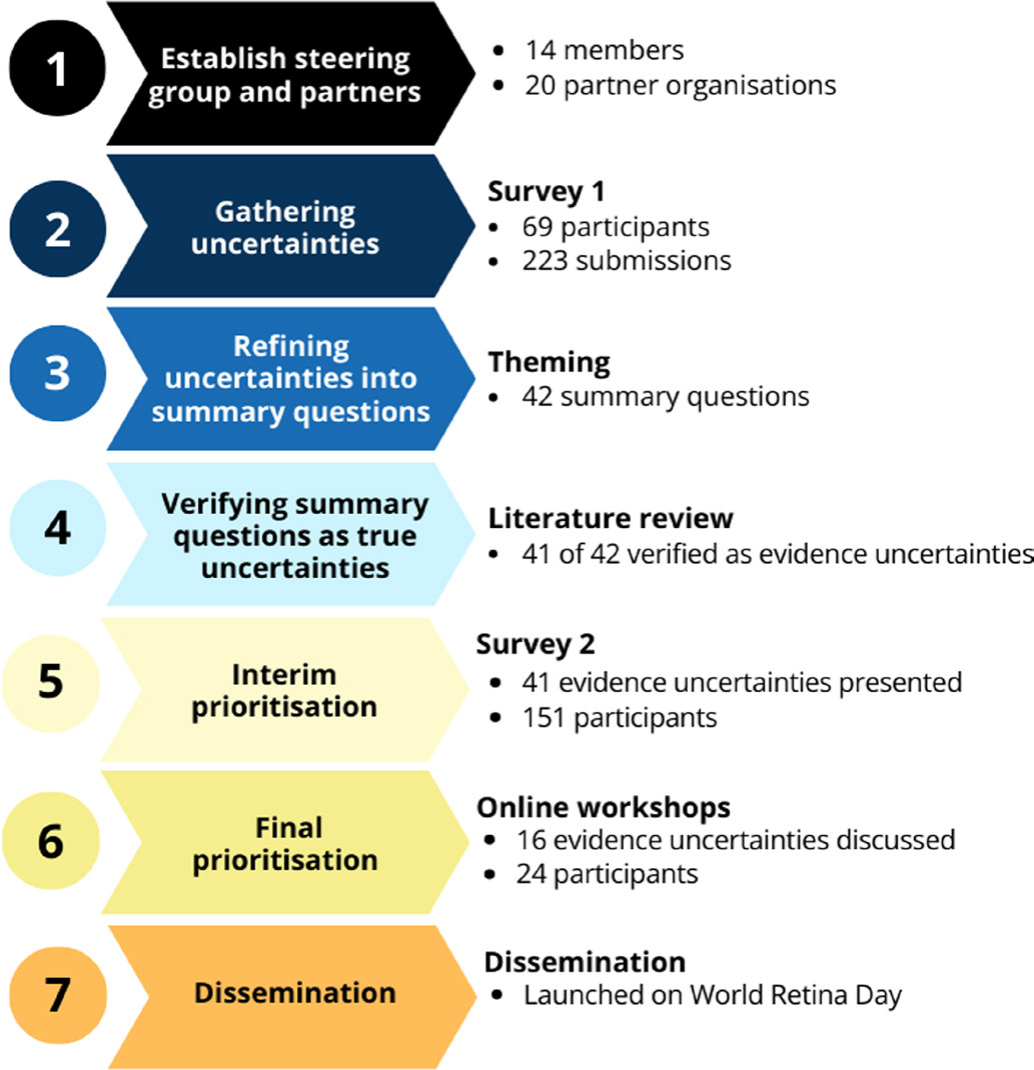
Summary of the key stages of our priority setting partnership.

The PSP process involved (1) establishing a steering group and partners; (2) defining the scope; (3) gathering evidence uncertainties; (4) refining uncertainties into summary questions; (5) verifying whether summary questions were true evidence uncertainties; (6) shortlisting evidence uncertainties through an interim prioritisation exercise and (7) finalising the top 10 priorities during live workshops. The integral final step is disseminating the priorities to maximise its success. See figure 1 for a summary of our PSP process.

### Patient and public involvement

Patients, caregivers and representatives of community support organisations have been central to our PSP from inception. Core to our chosen methodology, consumers were integrated throughout this research as partners. They co-developed the PSP scope, surveys and recruitment materials, and also supported recruitment, data interpretation and dissemination (including as co-authors).

### Establishing a steering group and partners

The PSP lead (EGR) was selected to drive the PSP process given their research and consumer engagement expertise. We established a steering group comprising 14 individuals who were responsible for overseeing the PSP process, guiding the accessibility of data collection, supporting recruitment, identifying partner organisations and disseminating findings. The steering group members included individuals impacted by an IRD (living with an IRD and/or a family member of someone with an IRD; n=5), health professionals/clinician-scientists (n=5), researchers (n=4) and representatives from community organisations (n=4). Several members represented more than one perspective (eg, an individual with lived experience and a researcher). No steering group member had prior experience with a PSP. Members with lived experience were remunerated at $A60 per hour of meeting attendance, with other members participating in-kind. Regular meetings were held online, chaired by an independent JLA advisor who was trained to guide the PSP process.

We invited organisations to be a partner at any time throughout the study if they were deemed to have established networks of individuals who may have an IRD and/or health professionals who care for individuals with an IRD. Overall, we had 20 partner organisations support recruitment and dissemination (e.g., Retina Australia, The Royal Australian and New Zealand College of Ophthalmologists).

### Defining scope

The steering group agreed that the scope of the PSP would include uncertainties (ie, unanswered research questions) related to the following:

- Prevention of an IRD (eg, carrier screening tests).
- Diagnosis of an IRD.
- Disease progression and control.
- Treatment and research into potential treatments.
- Epidemiology.
- Management of an IRD (including management of the physical, psychological, emotional, financial and social aspects of living with an IRD, or as a caregiver).

We excluded questions that focused on concomitant eye diseases (eg, glaucoma) or hearing loss that was not in reference to an IRD symptom or comorbidity.

Individuals were eligible to participate in the surveys and workshops if they:

- Had been diagnosed with an IRD.
- Were a caregiver of someone with an IRD (eg, parent and spouse).
- Were a health or supportive care professional with direct contact with individuals who have an IRD.

All participants were required to be 16 years or older and residing in Australia. Individuals with age-related macular degeneration as their only vision-related condition were ineligible to participate.

### Gathering evidence uncertainties

The steering group developed and tested Survey 1. Two additional individuals with lived experience of an IRD undertook user testing of the survey with alternate screen readers. The survey included several optional demographic items (eg, age range and postcode) and invited participants to submit up to five questions they would like answered about IRDs.

We launched the survey in September 2023, and it remained open for 8 weeks. The survey was available in multiple formats, including online (via Qualtrics), on paper (written and in Braille), over the phone, and via video call in Australian Sign Language (Auslan). The steering group and partner organisations disseminated the survey link through their professional networks (eg, newsletters, conferences and webinars), social media channels and via posters/flyers in private clinics. Our steering group reviewed responses after 4 weeks, which prompted further efforts to continue raising awareness about the survey to all networks and the development of a short video in Auslan, with an English voiceover, that gave an overview of what IRDs are and who to contact for study information.

### Refining uncertainties into summary questions

EGR downloaded and cleaned responses, confirmed the eligibility of participants, and then assigned uncertainties as in-scope or out-of-scope, which were confirmed by the steering group. We used basic descriptive statistics to summarise respondent demographics. EGR combined similar or duplicate questions and independently coded the submissions via Microsoft Excel. A subgroup of the steering group (one individual with lived experience [MP], one researcher and psychologist [KH] and EGR) then discussed the coding and categorised the submissions into draft summary questions. Questions were structured using the Population, Intervention, Comparison, Outcome format, where possible. The steering group discussed and further refined the draft summary questions until consensus was reached.

### Verifying evidence uncertainties

A subgroup of the steering group (one researcher [AGZ], three clinician-scientists [LA, AM and MS] and EGR) checked questions against the evidence to determine whether they were deemed answered or were an evidence uncertainty (ie, partially answered or not answered). We searched Cochrane Reviews, journal databases (MEDLINE, CINAHL, PsycINFO, Embase, PubMed and Google Scholar) and relevant guidelines from peak bodies that had been published between 2013 and 2024. Summary questions that were partially answered or not answered were classified as evidence uncertainties and included in the interim prioritisation survey. See online supplemental appendix A for further details.

### Interim prioritisation

Steering group members reviewed and tested the interim prioritisation survey, which was launched in March 2024. The survey remained open for 10 weeks. Survey administration (eg, paper and online delivery) and recruitment processes aligned with those of Survey 1. We also invited individuals to participate in Survey 2 during an IRD Patient and Family Engagement Day held in Sydney, Australia. Attendees were given a paper survey to complete on the day and a QR code for those who prefer to complete it online.

The survey included several optional demographic items and asked participants to vote for up to 10 of the evidence uncertainties they considered most important for researchers to address. For online administration, the evidence uncertainties were randomised for each participant. As with Survey 1, the steering group reviewed the representativeness of participants midway through recruitment to drive targeted recruitment efforts as needed (eg, to capture more respondents from a specific state).

Responses were downloaded, cleaned and analysed by EGR. Data were analysed separately for the four participant groups:

- Group A: individuals living with an IRD.
- Group B: caregivers and family members of someone with an IRD.
- Group C: lived experience (ie, groups A and B combined).
- Group D: health and supportive care professionals.

For each group, we tallied the votes and ranked the evidence uncertainties (with the #1 rank being the uncertainty with the highest number of votes). We then calculated a combined ranking by summing the rank score across Group C and Group D. This ensured that the perspectives of the lived experience and health professional groups were equally weighted. The steering group reviewed the rankings and confirmed the top-ranked uncertainties to take forward to final prioritisation.

### Final prioritisation workshops

We opened our expression of interest for the final workshops in June 2024 and accepted applications for 4 weeks. Participants were selected with the aim of achieving equal participation from individuals with lived experience and health professionals, as well as representation across age groups, gender, caregiver role and state. EGR telephoned the selected individuals to confirm their participation and discuss any accessibility requirements. Participants with lived experience were remunerated at $A40 per hour of workshop participation, as per the Health Consumers New South Wales guidelines (V.1).^21^

The final workshops were held online, over two half days in August 2024. Participants were sent the shortlisted uncertainties that had been determined in the interim prioritisation survey (Survey 2). As prework, participants were required to select their top and bottom three priorities. The two workshops followed the Nominal Group Technique, which involved two rounds of small group discussions on day 1 and a final round of small group discussions on day 2. On day 1, participants were divided into four small groups (each with a mix of individuals with lived experience and health professionals), along with a James Lind Alliance Facilitator. In Round 1, participants were asked to share their top and bottom three priorities with the group. The James Lind Alliance Facilitator arranged the questions on screen based on what their group deemed important versus less important. In Round 2, groups then discussed the priorities and established an initial ranking. At the end of day 1, the James Lind Alliance Facilitators averaged the rankings across each small group to obtain a combined ranking. On day 2, participants were allocated to new small groups. In Round 3, participants discussed and revised the combined ranking. The final rankings across each small group were again averaged to result in the final top 10 research priorities.

All workshop participants were invited to share feedback with the lead researcher about their experiences with the final prioritisation workshops via email or telephone call.

## Results

### Gathering evidence uncertainties

Overall, we received 223 in-scope submissions (n_out-of-scope_=21) from 69 eligible participants (n_ineligible_=6). Most participants were individuals living with an IRD or a caregiver/family member of someone with an IRD (n=54, 78%). Of these 54 individuals with lived experience, 50 specified the specific IRD type they were impacted by. The most common diagnoses were retinitis pigmentosa (n=20, 40%) and Usher syndrome (n=9, 18%). See table 1 for an overview of demographics.

**Table 1:** Demographics of participants across each priority setting partnership stage

	Gathering uncertainties (n=69)	Interim prioritisation (n=151)	Workshops (n=24)
Participant type			
Individual with an IRD	35*	92 (+ 7†)	9
Caregiver/family member	18	36 (+ 7†)	4
Health professional	17*‡	16	11
Gender			
Female	N/A	N/A	19
Male	N/A	N/A	5
Age			
Less than 18 years	3	N/A	0
18–24 years	7	N/A	1
25–34 years	12	N/A	5
35–44 years	16	N/A	3
45–54 years	12	N/A	6
55–64 years	12	N/A	4
65 years and above	3	N/A	4
Missing data	4	N/A	1
State			
New South Wales	19	28	13
Victoria	23	69	6
Queensland	6	18	3
Western Australia	11	9	1
South Australia	0	7	1
Tasmania	2	1	0
Northern Territory	0	0	0
Australian Capital Territory	0	0	0
Missing data	8	19	0
Remoteness			
Major city	46	24	15
Inner regional	1	37	3
Outer regional	14	71	6
Remote and very remote	0	0	0
Missing data	8	19	0
First language			
English	45	N/A	N/A
Other	3	N/A	N/A
Missing data	21	N/A	N/A
Aboriginal and/or Torres Strait Islander			
Yes	2	N/A	N/A
No	65	N/A	N/A
Missing data	2	N/A	N/A

Note: Demographics other than participant type were optional meaning that this data is not available for all participants.

* One individual indicated that they were both living with an IRD and a health professional caring for this cohort and thus counted in both groups.

† Seven individuals indicated that they were both living with an IRD and a caregiver of someone with an IRD.

‡ Specific type of health professional was collected only for the ‘Gathering Uncertainties’ survey and workshops. The survey and workshops are both representations from optometrists, genetic counsellors, clinical geneticists, orthoptists, social workers, deafblind consultants and support coordinators.

IRD, inherited retinal disease; N/A, not asked.

### Refining uncertainties into summary questions

We categorised 223 in-scope submissions into 42 summary questions across 10 domains. These domains included (1) caregivers and family, (2) diagnosis, (3) epidemiology, (4) healthcare and systems, (5) information and decision-making, (6) prevention, (7) research, (8) supportive care, (9) symptoms and comorbidities, and (10) treatment. The most common submissions related to treatment (n=50, 22%) and information and decision-making (n=44, 20%), with the least common relating to caregivers and family (n=3, 1%). See online supplemental appendix B for more details.

### Verifying evidence uncertainties

Upon review of the 42 summary questions, our steering group deemed 21 summary questions (50%) as evidence of uncertainty without needing to search the literature. One summary question was also deemed answered without needing to search the literature (*What is the likelihood of passing an IRD on to a biological child?*). For the remaining 20 summary questions, we determined that most were unanswered (n=17) with a few partially answered (n=3). The questions that were classified as partially answered had some existing relevant literature, but it was specific to one type of IRD only. This resulted in a total of 41 evidence uncertainties.

### Interim prioritisation

A total of 151 individuals responded to the interim prioritisation survey. Most participants were living with an IRD (n=92, 61%). Of the 135 participants (living with an IRD or caregiver/family member) who specified an IRD diagnosis, the most common diagnoses were retinitis pigmentosa (n=61, 45%) and Usher syndrome (n=18, 13%). See table 1.

Each of the 41 evidence uncertainties received at least one vote. Group C (lived experience group) had six of their top 10 included in the combined top 10, and Group D (health professional group) had seven included. The steering group initially agreed to take the top 13 of the combined rankings and an additional five uncertainties that were included in the top 10 of Group A (individuals with an IRD), Group B (caregiver/family members) or Group D (health professionals). Upon further discussion of these 18 uncertainties, the steering group removed two uncertainties—one because it was deemed answered (*How can IRDs be prevented?*) and one because it fell within the scope of another question that was being taken forward to the workshops (*How does exposure to sunlight and glare impact individuals with an IRD, and what strategies can be employed to minimise this impact?* was deemed inclusive in *What are the most effective ways to manage IRD symptoms?*). See online supplemental appendix C for more details.

### Final prioritisation workshops

We received 36 eligible expressions of interest for the final workshops. Of these, five withdrew their interest at follow-up, four were lost to follow-up, and three were not selected due to the need for representation across demographics (eg, state and age group) and/or limited rationale provided in their interest to participate. This resulted in a total of 24 workshop participants, of whom 13 (54%) had lived experience of an IRD. Of the nine participants who had an IRD, all indicated that they had minimal vision or were legally blind, and two individuals also had a hearing impairment. See table 1 for an overview of demographics.

Initially, participants described finding it challenging to identify the less important research areas from the list of 16 uncertainties, noting that they felt all were important. By the end of the workshops, the final top 10 research priorities had been identified. All four small groups had at least 8 of their top 10 priorities included in the combined top 10. Of note, all four small groups agreed that the #1 research priority was for treatments to safely prevent, slow down or stop vision loss. See online supplemental appendix D for the rankings for all 16 uncertainties taken through to workshops.

When presented with the final priorities, participants were invited to share their thoughts. Several commented that they were pleased to see a mix of different focus areas represented. A few participants shared that they were disappointed that a particular research area they were passionate about was not included in the top 10 and acknowledged the challenge in accommodating everyone’s priorities.

After the workshops, workshop participants received a copy of the final priorities via email and were invited to the final priority launch event. Several participants shared the value of being able to hear others’ perspectives and experiences.

The final top 10 research priorities, which fall under four domains, are shown in table 2.

**Table 2:** Final top 10 research priorities for inherited retinal disease

Rank	Domain	Research priorities
1	Treatment / cure	What treatments can safely prevent, slow down or stop vision loss that occurs for someone with an IRD?
2	Psychosocial well-being	What is the psychological impact of having an IRD, and what support is most effective?
3	Treatment / cure	What treatments can safely restore vision for someone with an IRD?
4	Psychosocial well-being	What are the information and psychosocial needs of individuals with an IRD and their families at diagnosis?
5	Health service delivery	What training and/or guidelines are needed for health professionals to provide optimal support for individuals with an IRD, from diagnosis and beyond?
6	Psychosocial well-being	What are the most effective ways to support carers and family members of an individual with an IRD?
7	Symptoms / disease progression	How do environmental and lifestyle factors influence IRD symptoms and disease progression?
8	Symptoms / disease progression	What are the most effective ways to manage IRD symptoms?
9	Health service delivery	How can a programme to detect IRDs as early in life as possible be implemented?
10	Symptoms / disease progression	What is the anticipated progression of vision loss for each IRD?

### Dissemination

The final research priorities were launched on World Retina Day (24 September 2024) during an in-person event at the New South Wales Parliament House, hosted by the Parliament Friends Group for Eye Health and Vision Care, and spotlighted nationally on the Australian Broadcasting Corporation’s flagship current affairs radio programme. We are now collaborating with partner organisations and relevant peak bodies to drive engagement of these research priorities, including advocating to government for funding allocation to these priority research areas.

## Discussion

This was the first James Lind Alliance PSP conducted in Australia for IRDs. The highest-ranked priority in our PSP was for treatments to safely prevent, slow down or stop vision loss, followed by the need to address the psychological impact of an IRD. The final priorities reflect the broad, unmet needs that the IRD community have around treatment/cure, symptoms and disease progression, psychosocial well-being and health service delivery. We used the James Lind Alliance PSP methodology to identify the top 10 research priorities for IRDs in Australia. This will provide the necessary evidence to inform future research agendas and funding priorities. Beyond funding bodies, researchers and research institutions as the target audience, these priorities are also relevant for health professionals, health services and community organisations regarding what matters most to the community. The robust methodology used and partnership with the IRD community have resulted in meaningful research priorities that are relevant now and well into the future. It has also provided valuable learnings for other researchers to undertake more meaningful consumer engagement and make their research more accessible for people with a vision impairment.^22^

Our priorities for treatment—either to prevent, slow down or stop vision loss (#1 priority) or to restore vision (#3 priority)—align with the top-ranked IRD priority for the 2012 UK PSP for sight loss.^18^ This is not unexpected given that there remains a dearth of treatments for IRDs internationally. In the past decade, basic science research and clinical trial endeavours have led to the first clinically available gene therapy, voretigene neparvovec-rzyl (Luxturna), specifically for biallelic *RPE65*-associated retinal dystrophy.^7^ Other than Luxturna, there are no other clinically available treatments to safely prevent vision loss or restore vision for someone with an IRD. The development of other gene therapies to address some of the 300+ known causative genes is ongoing, with numerous clinical trials open or in the pipeline. The genetic and phenotypic heterogeneity of IRDs, however, has also prompted the search for gene-agnostic therapies, including cell therapy,^23^ causative gene-agnostic gene therapies (eg, optogenetic therapies and photoswitch molecules) and electronic retinal implants.^24^ As clinical research progresses, multidisciplinary collaborations will be necessary to facilitate the successful implementation of trials and approved treatments when they become available in Australia.^25^ In line with the #5 priority, regarding training for health professionals, ongoing education for ophthalmologists who do not specialise in IRDs may be needed to facilitate access to genetic testing and counselling and appropriately counsel patients around novel therapeutics.^26 27^

Until treatments become available for all IRDs, research that investigates how to minimise disease burden will remain a priority. In our PSP, the IRD community highlighted the critical need to address the psychological impacts of an IRD for both individuals with an IRD and caregivers. The high prioritisation of these areas is likely to reflect the critical insights of those directly affected by IRDs, suggesting that these areas may be under-recognised and underaddressed. Individuals who have irreversible vision loss often experience poorer mental health and quality of life compared with the general population.^9^,^1228^ From the point of diagnosis, individuals can experience significant distress, anticipatory grief and a sense of hopelessness.^31^ Community-based mental health professionals (eg, psychologists) and primary health professionals (eg, general practitioners) may not have the knowledge to provide the support that is uniquely required for patients diagnosed with an IRD and its associated vision loss. Often, this leaves ophthalmologists responsible for providing this support. RANZCO’s 2020 ‘Guidelines for the assessment and management of patients with inherited retinal degenerations’ highlight that ‘*psychological support is also necessary so that individuals and families can deal with the emotional stress and sometimes uncertainty associated with an inherited retinal disease*’.^6^ The Australian ophthalmology workforce currently has minimal time (with ~3.9 full-time equivalent clinicians per 100 000 population)^32^ and little practical guidance to address the substantial and ongoing psychosocial impact of living with an IRD.^27 33^ The lack of appropriate support and information provision can then exacerbate feelings of distress. Beyond the public healthcare system, community organisations also play a crucial role in addressing some psychosocial and information needs. Increased awareness of organisations and available services is critical for ensuring equitable access from the point of diagnosis.

Alongside progress towards a treatment or cure, participants also prioritised research into environmental and lifestyle factors that may modulate disease burden.^34^ For example, in Stargardt (*STGD1*) disease, a high intake of vitamin A is suspected to be a risk factor for progression.^35^ Natural history studies are critical in understanding disease progression and the role of environmental and lifestyle factors. They can also facilitate the exploration of genetic-phenotypic correlations and thus identify potential therapeutic targets. There are major IRD treatment centres in public teaching hospitals in each Australian state, and each has an associated state-based registry. Good collaboration exists between Australian centres to contribute to natural history studies. However, given the rarity of individual genotypes, international collaborations would also be valuable.^27^

To address many of the priority areas, substantial time, funding, skills and capacity are needed. Further, research areas that were not explicitly identified as a top 10 priority may be foundational to progress in the priority areas. While the PSP process facilitates the identification and ranking of research priorities, research areas are often not independent. One such example is improved access to equitable genetic testing and genetic counselling. This research area was not included in our top 10 research priorities. However, genetic testing is a prerequisite to support ophthalmic investigations, guide the development of new therapies, identify those eligible for new genetic therapies and educate families about their condition.^36^ The RANZCO IRD management guidelines state that clinical genetic testing is part of routine care. Genetic testing is freely available in Australian public hospitals via clinical genetics services. However, access to such testing may still be inequitable and state-dependent in Australia, with limited genetic counselling services to guide families through the complexities of the process.^27^ In addition to local access issues, there may also be misunderstandings about out-of-pocket costs, identified as a barrier to testing for the IRD community internationally.^37^

## Conclusions

We undertook a James Lind Alliance PSP to identify the top 10 research priorities for IRDs in Australia. The wide scope of priorities across treatment, symptoms/progression, psychosocial well-being and health service delivery highlights the need for a multidisciplinary, systems approach to IRD research and care. Driving research that aligns with the priorities set by individuals impacted by IRDs will accelerate the translation of meaningful research into real-world benefits. Funding bodies, researchers, clinicians and community organisations collectively have the responsibility of addressing these priorities.^38^

## Supplementary material

10.1136/bmjopen-2025-100301 online supplemental file 1

## Data Availability

Data are available upon reasonable request.
