# Supplementary material for: Australian research priorities for inherited retinal diseases: a James Lind Alliance priority setting partnership
Source: BMJ Open. 2025 Sep 30;15(9):e100301. doi: 10.1136/bmjopen-2025-100301 (PMC12519664; doi:10.1136/bmjopen-2025-100301)
Supplement: online supplemental file 1 [file bmjopen-15-9-s001.docx]

**Appendix A. Evidence checking process**

| **Sources** | - Cochrane Reviews, using keywords specific to each question. - Database searches on MEDLINE, CINAHL, PsycINFO, Embase, PubMed, Google Scholar. - Guidelines:   - Royal Australia and New Zealand College of Ophthalmologists (RANZCO)   - American Academy of Ophthalmology (USA)   - Royal College of Ophthalmology (UK)   - Optometry Australia (Australia)   - British College of Optometrists (UK)   - American Academy of Optometry (USA)   - Human Genetics Society of Australia   - American College of Medical Genetics   - Australian and New Zealand Clinical Trials Registry (ANZCTR) and clinicaltrials.gov. |
| --- | --- |
| **Search terms for Cochrane and database searches** | - systematic review* OR meta analys* OR meta-analys* OR metaanaly* OR meta synthes* OR meta-synthes* OR review* OR guideline*) AND - (inherited retinal disease OR retinitis pigmentosa OR usher OR vision loss OR stargardt OR cone dystrophy OR vision impairment OR blind OR retinal dystrophy) AND - *[additional search terms to reflect the concepts/focus of each question]* |
| **Parameters** | - published from 2013 to 2024 - limited to publication in English in a peer-reviewed journal or peak body in the case of guidelines. |

**Categorisation of evidence**

1. **Answered:** Reliable, up-to-date systematic reviews, meta-analyses or clinical guidelines have been published.
2. **Partially answered:** Relevant, reliable and up-to-date systematic reviews, meta-analyses and evidence-based guidelines but do not address continuing questions; a current clinical trial but not exhaustive enough to answer the question, or a relevant systematic review but not up-to-date (i.e., published before 2013).
3. **Not answered:** No relevant systematic reviews, meta-analyses or clinical guidelines identified.

**Appendix B. Themes of unanswered questions submitted, with example summary question**

| **Theme** | **Example summary questions** | **Number of submissions by theme** |
| --- | --- | --- |
| Treatment | What treatments can safely restore vision? | 50 |
| Information and decision-making | In communicating about clinical trials and research updates, what methods are most effective in conveying information to individuals with an IRD and families? | 44 |
| Symptoms and comorbidities | What additional health conditions are associated with each IRD? | 39 |
| Healthcare and system | How can coordinated support from relevant organisations and services (e.g., health services, NDIS) be provided? | 34 |
| Epidemiology | What is the anticipated progression of vision loss for each IRD? | 16 |
| Diagnosis | How can the pathway to diagnosis be improved so that they are accurate and efficient? | 14 |
| Prevention | How can IRDs be prevented? | 13 |
| Supportive care | What is the psychological impact of having an IRD, and what support is most effective? | 6 |
| Research | How can artificial intelligence (AI) be used to enhance and expedite research into IRDs? | 4 |
| Caregivers and family | What are the most effective ways to support carers and family members of an individual with an IRD? | 3 |

**Appendix C. Interim prioritisation rankings by participant group, and overall**

| **Interim rank** | **Evidence uncertainties** | **Participant group rank** | | |
| --- | --- | --- | --- | --- |
|  |  | **w/ IRD**  **(n=99)** | **Caregiver (n=42)** | **HP (n=17)** |
| 1 | What treatments can safely prevent, slow down or stop vision loss? *^a^* | 1 | 1 | 1 |
| 2 | What treatments can safely restore vision? *^a^* | 2 | 2 | 4 |
| 3 | What is the anticipated progression of vision loss for each IRD? *^a^* | 4 | 3 | 7 |
| 4 | What is the psychological impact of having an IRD, and what support is most effective? *^a^* | 5 | 4 | 10 |
| 5 | How can equitable access to genetic testing and genetic counselling be implemented across Australia? *^a^* | 17 | 20 | 2 |
| 6 | What are the most effective ways to support carers and family members of an individual with an IRD? *^a^* | 18 | 5 | 5 |
| 7 | What training and/or guidelines are needed for health professionals to provide optimal support for individuals with an IRD, from diagnosis and beyond? *^a^* | 14 | 21 | 6 |
| 8 | What is an effective treatment that is not gene-specific? *^a^* | 6 | 8 | 15 |
| 9 | How can artificial intelligence (AI) be used to enhance and expedite research into IRDs? *^a^* | 10 | 7 | 11 |
| 10 | What are the most effective ways to manage IRD symptoms (e.g., low vision at night)? *^a^* | 11 | 10 | 16 |
| 11 | How can coordinated support from relevant organisations and services (e.g., health services, NDIS) be successfully implemented? *^a^* | 20 | 26 | 3 |
| 12 | How do environmental and lifestyle factors influence symptoms and disease progression? *^a^* | 3 | 6 | 21 |
| 13 | What are the information and psychosocial needs of individuals with an IRD and their families at diagnosis? *^a^* | 21 | 15 | 8 |
| 14 | How can the latest research be effectively communicated to health professionals caring for IRD patients? | 19 | 17 | 12 |
| 15 | How can IRDs be prevented? ^b^ | 8 | 11 | 26 |
| 16 | What is the best way to facilitate peer-support networks for individuals with an IRD? *^a^* | 23 | 38 | 9 |
| 17 | What is the impact of genetic testing for an IRD on patients and families, and how does this information impact disease management and patient decision-making? | 27 | 24 | 13 |
| 18 | In communicating about clinical trials and research updates, what methods are most effective in conveying information to patients and families? | 15 | 13 | 22 |
| 19 | What are the biological mechanisms that lead to vision loss for each IRD? | 22 | 18 | 17 |
| 20 | How does exposure to sunlight and glare impact individuals with an IRD, and what strategies can be employed to minimise this impact? ^c^ | 7 | 22 | 32 |
| 21 | How can a program to detect IRDs as early in life as possible be implemented? *^a^* | 9 | 12 | 33 |
| 22 | How do individuals with an IRD visually perceive their surroundings? | 26 | 16 | 23 |
| 23 | How can the pathway to diagnosis be improved so that they are accurate and efficient? | 29 | 14 | 18 |
| 24 | How can training and/or guidelines for health professionals caring for individuals with an IRD be successfully implemented? | 30 | 25 | 14 |
| 25 | What are the optimal ways to measure an individual's level of vision impairment, specifically for IRDs? *^a^* | 12 | 9 | 34 |
| 26 | How does early detection and planning impact disease outcomes? | 16 | 40 | 27 |
| 27 | In communicating about genetics of IRDs, genetic testing and risk of disease inheritance, what methods are most effective in conveying information to patients and families? | 31 | 27 | 19 |
| 28 | What are the risks of surgery for other eye conditions that may co-occur with an IRD? | 13 | 35 | 35 |
| 29 | What is the benefit and burden of follow-up care after a diagnosis of an IRD? | 25 | 36 | 28 |
| 30 | How do clinical, genetic and demographic factors influence the effectiveness and risks of different treatment options? | 32 | 23 | 29 |
| 31 | What the most effective ways to manage and/or prevent related health conditions that may co-occur with an IRD? | 28 | 30 | 30 |
| 32 | What factors influence patient and caregiver decisions about IRD treatment options? | 37 | 29 | 24 |
| 33 | In communicating general health information and support resources, what methods are most effective in conveying information to patients and families? | 38 | 33 | 20 |
| 34 | What is the impact of having an IRD with additional health conditions on quality of life? | 40 | 30 | 25 |
| 35 | How can public awareness regarding IRDs and related health conditions be increased? | 24 | 28 | 40 |
| 36 | What is the impact of caring for a child with an IRD on quality of life? | 39 | 19 | 31 |
| 37 | What additional health conditions are associated with each IRD? | 36 | 34 | 36 |
| 38 | What is the physiological and psychological impact of restoring sight? | 33 | 39 | 37 |
| 39 | What is the physical impact of being a carrier of an IRD? | 35 | 32 | 38 |
| 40 | What other eye-related health conditions (e.g., cataracts) are associated with each IRD? | 34 | 41 | 39 |
| 41 | How common are IRDs and the various subtypes? | 41 | 37 | 41 |

*Note. w/ IRD = individual living with an IRD; HP = health professional; a lower rank indicates a higher number of votes*

**Appendix D. Workshop final small group rankings and collective overall rankings**

| **Overall ranking** | **Research priorities** | **Small group rankings** | | | |
| --- | --- | --- | --- | --- | --- |
|  |  | **G1** | **G2** | **G3** | **G4** |
| **1** | What treatments can safely prevent, slow down or stop vision loss that occurs for someone with an IRD? | 1 | 1 | 1 | 1 |
| **2** | What is the psychological impact of having an IRD, and what support is most effective? | 4 | 3 | 3 | 3 |
| **3** | What treatments can safely restore vision for someone with an IRD? | 5 | 2 | 2 | 8 |
| **4** | What are the information and psychosocial needs of individuals with an IRD and their families at diagnosis? | 2 | 4 | 13 | 2 |
| **5** | What training and/or guidelines are needed for health professionals to provide optimal support for individuals with an IRD, from diagnosis and beyond? | 6 | 11 | 4 | 4 |
| **6** | What are the most effective ways to support carers and family members of an individual with an IRD? | 10 | 5 | 5 | 6 |
| **7** | How do environmental and lifestyle factors influence IRD symptoms and disease progression? | 3 | 6 | 12 | 7 |
| **8** | What are the most effective ways to manage IRD symptoms? | 9 | 8 | 8 | 10 |
| **9** | How can a program to detect IRDs as early in life as possible be implemented? | 8 | 7 | 7 | 13 |
| **10** | What is the anticipated progression of vision loss for each IRD? | 11 | 9 | 9 | 9 |
| **11** | How can equitable access to genetic testing for IRDs and genetic counselling be implemented across Australia? | 7 | 12 | 6 | 15 |
| **12** | How can coordinated IRD support from relevant organisations and services (e.g., health services, NDIS) be successfully implemented? | 12 | 10 | 11 | 12 |
| **13** | What is the best way to facilitate peer-support networks for individuals with an IRD? | 13 | 15 | 15 | 5 |
| **14** | What is an effective treatment for IRDs that is not gene-specific? | 14 | 13 | 14 | 14 |
| **15** | What are the optimal ways to measure an individual's level of vision impairment, specifically for IRDs? | 16 | 14 | 16 | 11 |
| **16** | How can artificial intelligence be used to enhance and expedite research into IRDs? | 15 | 16 | 10 | 16 |

*Note. Each small group consisted of 5-6 participants with representation from individuals who have an IRD, caregivers and health professionals. The overall ranking was obtained by averaging the small group ranking, with a lower number rank indicating a higher priority. In cases where two uncertainties received the same average ranking, the final order was determined using the midrange of their respective ranks.*
