# Supplementary material for: Australian research priorities for inherited retinal diseases: a James Lind Alliance priority setting partnership
Source: BMJ Open. 2025 Sep 30;15(9):e100301. doi: 10.1136/bmjopen-2025-100301 (PMC12519664; doi:10.1136/bmjopen-2025-100301)
Supplement: Supplementary file 2 [file bmjopen-2025-100301.reviewer_comments.pdf]

PEER REVIEW HISTORY

BMJ Open publishes all reviews undertaken for accepted manuscripts. Reviewers are asked to complete a checklist review form and are provided with free text boxes to elaborate on their assessment. These free text comments are reproduced below.

ARTICLE DETAILS

Title (Provisional)

Australian research priorities for inherited retinal diseases: a James Lind Alliance Priority Setting Partnership

Authors

Robertson, Eden G; Hetherington, Kate; Prain, Meredith; Ma, Alan; Ayton, Lauren; Jamieson, Robyn; Shepard, Emily; Boyd, Leighton; Hall, Julia; Boyd , Rosemary; Karandrews, Sally; Feller, Hollie; Simunovic , Matthew; Grigg, John; Yamamoto , Kanae; Wakefield, Claire; Gonzalez-Cordero, Anai

---

VERSION 1 - REVIEW

Reviewer **1**

Name **Ying, Gui-shuang**

Affiliation **University of Pennsylvania Perelman School of Medicine, Ophthalmology**

Date **13-Jul-2025**

COI **None**

This is a well-conducted study. The paper was well-written. I only have one comment to improve the presentation of results, specifically, it will be informative to show the frequency of votes received from 24 workshop participants on the top 10 research priorities from list of 16 uncertainties.

Also, a flow diagram on the whole study design will be very helpful to demonstrate the whole study process.

Reviewer **2**

Name **Falsini, Benedetto**

Affiliation **Fondazione Policlinico Universitario Agostino Gemelli IRCCS, UOC Oftalmologia**

Date **01-Aug-2025**

COI **None**

In this study, the authors aimed to identify the top 10 Australian research priorities for IRDs to ultimately facilitate more meaningful and potentially cost-effective research.They found that the top 10 priorities represented: i) treatment/cure; ii) symptoms and disease progression; iii) psycho-social well being; and iv) health service delivery. The study is well conducted and the results accurately presented. However, the conclusions are quite obvious, being represented mainly by treatment cure as a top priority. I do not see much value and novel implications in the results of this survey, because it is already known world-wide that treatment/cure is a top priority, especially in pediatric patients.

VERSION 1 - AUTHOR RESPONSE

Reviewer 1

1. I only have one comment to improve the presentation of results, specifically, it will be informative to show the frequency of votes received from 24 workshop participants on the top 10 research priorities from list of 16 uncertainties.

Author response: Thank you for this recommendation. We have chosen to include this information as an Appendix D given the overlap in content provided in Figure 2. Appendix D presents the overall ranking of the 16 uncertainties discussed during the workshops, along with the small group rankings used to calculate these overall rankings. As per the James Lind Alliance methodology, we recorded participant rankings in our workshops rather than vote frequencies; therefore, we are unable to report on the latter.

2. A flow diagram on the whole study design will be very helpful to demonstrate the whole study process.

Author response: Our original submission included Figure 1, which outlines the overall study design and data collection process. To support readers, this is introduced early in the 'Methods and Materials' section. Should the editor have any specific suggestions to revise Figure 1, we would be pleased to consider them.

Reviewer 2

1. The conclusions are quite obvious, being represented mainly by treatment cure as a top priority. I do not see much value and novel implications in the results of this survey, because it is already known world-wide that treatment/cure is a top priority, especially in pediatric patients.

Author response: Thank you for your feedback. We acknowledge that the prioritisation of treatment to restore sight and prevent vision loss is a well-

established concern, given the current lack of treatment options for inherited retinal diseases. This has been mentioned in our Discussion, Paragraph 2. While the emphasis on treatment was anticipated, the strong prioritisation of research into the psychological impact of IRDs and support for carers is more novel. The high prioritisation of these areas is likely to reflect the critical insights of those directly affected by IRDs, suggesting that these areas may be under-recognised and under-addressed. As such, our findings help to bring them into sharper focus for future research and policy efforts. We have added in a sentence to our Discussion that elaborates on this:

> *"In our PSP, the IRD community highlighted the critical need to address the psychological impacts of an IRD, for both individuals with an IRD and caregivers. The high prioritisation of these areas is likely to reflect the critical insights of those directly affected by IRDs, suggesting that these areas may be under-recognised and under-addressed."*

The novelty of our study also lies in the use of the structured James Lind Alliance Priority Setting Partnership. To our knowledge, this is the first priority setting exercise (of any kind) conducted with the inherited retinal disease community, and that brings together such a diverse range of stakeholder perspectives, including those with lived experience, across all levels of the study. In addition to identifying research priorities, our study adds value by highlighting the importance of meaningful consumer engagement throughout the research process. We have added in a sentence to our Discussion that elaborates on this:

"The robust methodology used and partnership with the IRD community has resulted in meaningful research priorities, that are relevant now and well into the future. It has also provided valuable learnings for other researchers to undertake more meaningful consumer engagement and make their research more accessible for people with a vision impairment.[21]"

We hope these revisions and clarifications address the feedback provided and strengthen the manuscript. Thank you again for your time and consideration. We remain grateful for the opportunity to contribute to *BMJ Open*.
